# Tonotopic representation of loudness in the human cortex

**DOI:** 10.1016/j.heares.2016.11.015

**Published:** 2017-02

**Authors:** Andrew Thwaites, Josef Schlittenlacher, Ian Nimmo-Smith, William D. Marslen-Wilson, Brian C.J. Moore

**Affiliations:** a Department of Psychology, University of Cambridge, Cambridge, UK; b MRC Cognition and Brain Sciences Unit, Cambridge, UK

**Keywords:** Tonotopy, Magnetoencephalography, Loudness, Temporal integration, Model expression, Entrainment, CF, characteristic frequency, DLS, dorso-lateral sulcus, EMEG, electro- and magneto-encephalographic, HG, Heschl's gyrus, KID, Kymata identifier

## Abstract

A prominent feature of the auditory system is that neurons show tuning to audio frequency; each neuron has a characteristic frequency (CF) to which it is most sensitive. Furthermore, there is an orderly mapping of CF to position, which is called tonotopic organization and which is observed at many levels of the auditory system. In a previous study (Thwaites et al., 2016) we examined cortical entrainment to two auditory transforms predicted by a model of loudness, instantaneous loudness and short-term loudness, using speech as the input signal. The model is based on the assumption that neural activity is combined across CFs (i.e. across frequency channels) before the transform to short-term loudness. However, it is also possible that short-term loudness is determined on a channel-specific basis. Here we tested these possibilities by assessing neural entrainment to the overall and channel-specific instantaneous loudness and the overall and channel-specific short-term loudness. The results showed entrainment to channel-specific instantaneous loudness at latencies of 45 and 100 ms (bilaterally, in and around Heschl's gyrus). There was entrainment to overall instantaneous loudness at 165 ms in dorso-lateral sulcus (DLS). Entrainment to overall short-term loudness occurred primarily at 275 ms, bilaterally in DLS and superior temporal sulcus. There was only weak evidence for entrainment to channel-specific short-term loudness.

## Highlights

• The latency of cortical entrainment to various aspects of loudness was assessed.
• For channel-specific instantaneous loudness the latencies were 45 and 100 ms.
• For overall instantaneous loudness the latency was 165 ms.
• For overall short-term loudness the latency was 275 ms.
• Entrainment to channel-specific short-term loudness was weak.

## 1 Introduction

The loudness of a sound is a subjective attribute corresponding to the impression of its magnitude. Glasberg and Moore (2002) proposed that there are two aspects of loudness for time-varying sounds such as speech and music. One is the short-term loudness, which corresponds to the loudness of a short segment of sound such as a single word in speech or a single note in music. The second is the long-term loudness, which corresponds to the overall loudness of a relatively long segment of sound, such as a whole sentence or a musical phrase. Glasberg and Moore (2002) proposed a model in which transformations and processes that are assumed to occur at relatively peripheral levels in the auditory system (i.e. the outer, middle, and inner ear) are used to construct a quantity called “instantaneous loudness” that is not available to conscious perception, although it appears to be represented in the brain (Thwaites et al., 2016). At later stages in the auditory system the neural representation of the instantaneous loudness is assumed to be transformed into the short-term loudness and long-term loudness, via processes of temporal integration.

In a previous study (Thwaites et al., 2016), we investigated whether the time-varying instantaneous loudness or short-term loudness predicted by the loudness model of Glasberg and Moore (2002) is ‘tracked’ by cortical current, a phenomenon known as cortical entrainment. The extent of cortical entrainment was estimated from electro- and magneto-encephalographic (EMEG) measures of cortical current, recorded while normal-hearing participants listened to continuous speech. There was entrainment to instantaneous loudness bilaterally at 45 ms, 100 ms and 165 ms, in Heschl's gyrus (HG), dorso-lateral sulcus (DLS) and HG, respectively. Entrainment to short-term loudness was found in both the DLS and superior temporal sulcus at 275 ms. These results suggest that short-term loudness is derived from instantaneous loudness, and that this derivation occurs after processing in sub-cortical structures.

Missing from this account is how overall instantaneous loudness is constructed from the information that flows from the cochlea. A prominent feature of the auditory system is that neurons show tuning to audio frequencies; each neuron has a characteristic frequency (CF) to which it is most sensitive. This is a consequence of the filtering that occurs in the cochlea, which decomposes broadband signals like speech and music into multiple narrow frequency channels. The information in each channel is transmitted, in parallel, along the afferent fibers making up the auditory nerve (Helmholtz, 1863, Fuchs, 2010, Meyer and Moser, 2010, von Bekesy, 1949), and information encoded in these channels reaches the cortex in separate locations (Saenz and Langers, 2014). Furthermore, there is an orderly mapping of CF to position, which is called tonotopic organization and which is observed at many levels of the auditory system (Palmer, 1995).

The model of Glasberg and Moore (2002) is based on the assumption that neural activity is combined across CFs, i.e. across frequency channels, before the transformation to short-term loudness. It is not known where in the brain such a combination might occur, but if there is cortical entrainment to channel-specific activity, the model leads to the prediction that this entrainment should occur with a shorter latency than for short-term loudness. In this study, we aimed to test this, by assessing neural entrainment to the instantaneous loudness predicted by the loudness model for nine frequency sub-bands, hereafter called “channels”, spanning the range from low to high frequencies.

**Fig. 1:**
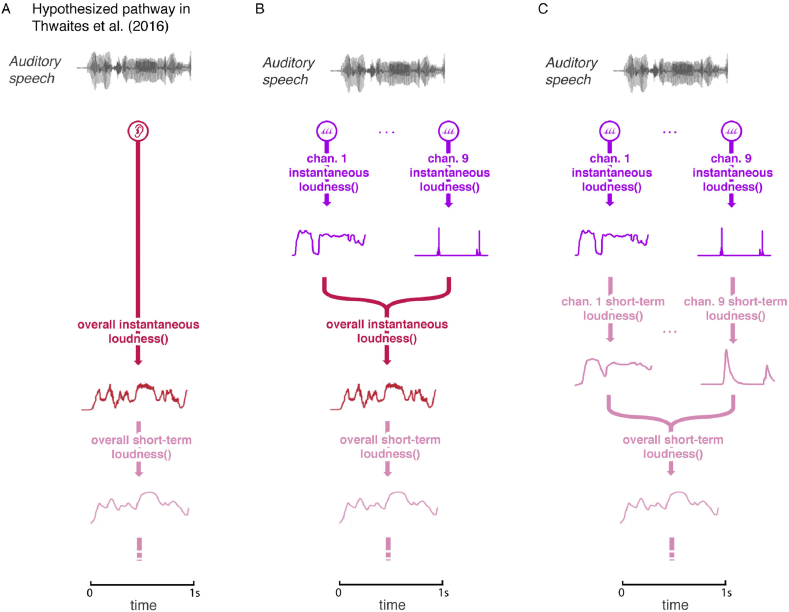
Example of the stimulus and model predictions. A. The hypothesized pathway in Thwaites et al. (2016), with the predicted activity for the first 1-s of the stimulus. B. The hypothesized pathway when *channel-specific instantaneous loudnesses* (purple) are added to the model. C. The hypothesized pathway when *channel-specific short-term loudnesses* (pink) are calculated as a prerequisite before the overall short-term loudness. (For interpretation of the references to colour in this figure legend, the reader is referred to the web version of this article.)

The sequence of transforms assumed in the model of Glasberg and Moore (2002) (channel-specific instantaneous loudness, followed by overall instantaneous loudness, followed by overall short-term loudness, outlined in Fig. 1) is not the only plausible sequence leading to the neural computation of overall short-term loudness. Short-term loudness may instead be derived separately for each channel, and these short-term loudness estimates may then be combined across channels to give the overall short-term loudness, as assumed in the models of Chalupper and Fastl (2002) and Moore et al. (2016). To test this alternative, we created a modified version of the model in which the short-term loudness was calculated separately for each channel and we assessed whether there was cortical entrainment to the channel-specific short-term loudness values. If neural responses are combined across CFs before the derivation of short-term loudness, then there might be cortical entrainment to the channel-specific instantaneous loudness values but not to the channel-specific short-term loudness values. If the short-term loudness is derived separately for each CF and then the short-term loudness estimates are combined across CFs, then cortical entrainment might be observed both to the channel-specific instantaneous loudness values and to the channel-specific short-term loudness values. Furthermore, the cortical entrainment to the channel-specific short-term loudness values should occur earlier in time than the cortical entrainment to the overall short-term loudness values.

In addition to standard graphic representations, an interactive representation of this study's results can be viewed on the online Kymata Atlas (http://kymata.org). For easy reference, each model in this paper (referred to as a ‘function’ in Kymata) is assigned a *Kymata ID* [KID].

## 2 Defining candidate models

To measure cortical entrainment, some constraints must be imposed on the models/transforms that can be tested. Any model that takes a time-varying signal as input and produces a time-varying signal as output can be used, with function *f*() characterizing the mechanism by which the information (in this case the acoustic waveform) is transformed before it produces cortical entrainment. Thus, if both input x_1_, …,x_m_ and output y_1,_ …,y_n_ are of duration *t*, the model takes the form:

(1) *f*(x_1_,x_2_,x_3_, …,x_t_) = (y_1_,y_2_,y_3_, …,y_t_),

where *f*() is bounded by a set of formal requirements (Davis et al., 1994) and a requirement that y_i_ cannot be dependent on any x_k_ where k > i (this last requirement avoids hypothesizing a non-causal *f*() where a region can express an output before it has the appropriate input).

Previously, three ‘auditory magnitude’ models were tested using the same dataset as the one used here: instantaneous loudness, short-term loudness, and the Hilbert envelope (KIDs: QRLFE, B3PU3 and ZDSQ9, respectively). The instantaneous loudness model (Moore et al., 1997, Glasberg and Moore, 2002) and short-term loudness model (Glasberg and Moore, 2002) represent successive transformations that approximate physiological processing in the peripheral and central auditory system. The third model, the Hilbert envelope model, was uninformed by physiological processing, and was included as a naïve comparison model.

The loudness model of Glasberg and Moore (2002) is based on a series of stages that mimic processes that are known to occur in the auditory system. These stages are: (1) A linear filter to account for the transfer of sound from the source (e.g. a headphone or loudspeaker) to the tympanic membrane; (2) A linear filter to account for the transfer of sound pressure from the tympanic membrane through the middle ear to pressure difference across the basilar membrane within the cochlea; (3) An array of level-dependent bandpass filters, resembling the filters that exist within the cochlea (Glasberg and Moore, 1990). These filters are often called the “auditory filters” and they provide the basis for the tonotopic organization of the auditory system; (4) A compressive nonlinearity, which is applied to the output of each auditory filter, resembling the compression that occurs in the cochlea (Robles and Ruggero, 2001).

The model of Glasberg and Moore (2002) is based on the assumption that the compressed outputs of the filters are combined to give a quantity proportional to instantaneous loudness. The moment-by-moment estimates of instantaneous loudness are then smoothed over time to give the short-term loudness. However, here we also consider a model based on the assumption that the smoothing over time occurs on a channel-specific basis, and that the overall short-term loudness is derived by summing the short-term specific loudness values across channels. The estimates of overall instantaneous loudness and channel-specific instantaneous loudness were updated every 1 ms. Examples of the structure of these models (and the models' predicted activity) are shown in Fig. 1.

We chose to use nine channels, each 4 Cams wide on the ERB_N_-number scale (Glasberg and Moore, 2002, Moore, 2012). The ERB_N_-number scale is a perceptually relevant transformation of the frequency scale that corresponds approximately to a scale of distance along the basilar membrane. The equation relating ERB_N_-number in Cams to frequency, *f*, in Hz is (Glasberg and Moore, 1990):

(2) ERB_N_-number = 21.4 log_10_(0.00437*f* + 1)

The normal auditory system uses channels whose bandwidths are approximately 1-Cam wide (Moore, 2012). However, cortical entrainment to the activity in 1-Cam wide channels might be very weak, because each channel contains only a small amount of energy relative to the overall energy. We used 4-Cam wide channels so as to give reasonable frequency specificity while avoiding channels that were so narrow that they would contain little energy and therefore lead to very “noisy” cortical representations. A second reason for not using 1-Cam wide channels is that the magnitudes of the outputs of closely spaced frequency channels in response to speech are highly correlated, whereas more widely spaced channels show lower correlations in their outputs (Crouzet and Ainsworth, 2001). The analysis method used in this paper does not work well when the models that are being compared give highly correlated outputs. Analyses using narrower channels may be possible in future studies if methods can be found for reducing the noise in the measured cortical activation.

In total, twenty-one candidate models were tested, each based on a different transform of the input signal. These were: the overall instantaneous loudness model; nine channel-specific instantaneous loudness models; the short-term loudness model; nine channel-specific short-term loudness models; and the Hilbert envelope model. The following sections outline these models, with the exception of the Hilbert envelope model, which is described in Thwaites et al. (2016).

### 2.1 Overall instantaneous loudness (KID: QRLFE)

The specific loudness is a kind of loudness density. It is the loudness derived from auditory filters with CFs spanning a 1-Cam range. To obtain the overall instantaneous loudness, the specific instantaneous loudness is summed for filters with CFs between 1.75 and 39.0 Cams (corresponding to 47.5 Hz and 15,108 Hz, respectively), as described by Glasberg and Moore (2002) and Thwaites et al. (2016). To achieve high accuracy, the CFs of the filters are spaced at 0.25-Cam intervals, and the sum is divided by 4.

### 2.2 Channel-specific instantaneous loudness

The operation of the model for the calculation of channel-specific instantaneous loudness can be summarized using an equation that characterizes spectral integration. For each channel employed here, the specific loudness values were summed over a range of 4 Cams. For example, channel 4 used CFs between 13.75 and 17.5 Cam, corresponding to frequencies from 766 to 1302 Hz. Table 1 gives the center frequency of each channel in Hz and in Cams. The compressed outputs of all auditory filters with center frequencies within the span of a given channel were summed to give the channel-specific instantaneous loudness.

### 2.3 Overall short-term loudness (KID: B3PU3)

The overall short-term loudness was calculated from the overall instantaneous loudness, as described by Glasberg and Moore (2002) and Thwaites et al. (2016), by taking a running average of the instantaneous loudness. The averager resembled the operation of an automatic gain control system with separate attack and release times.

### 2.4 Channel-specific short-term loudness values

The channel-specific short-term loudness values were calculated in the same way as for the overall short-term loudness, except that the averager was applied to the channel-specific instantaneous loudness values. The KIDs of the channel-specific short-term loudness values are PH5TU, 9T98H, U5CM6, EFFZT, YRKEG, K3NT5, 5DS7S, PPVLF, and 9ZYZ4, for channels 1–9, respectively.

## 3 The analysis procedure

**Fig. 2:**
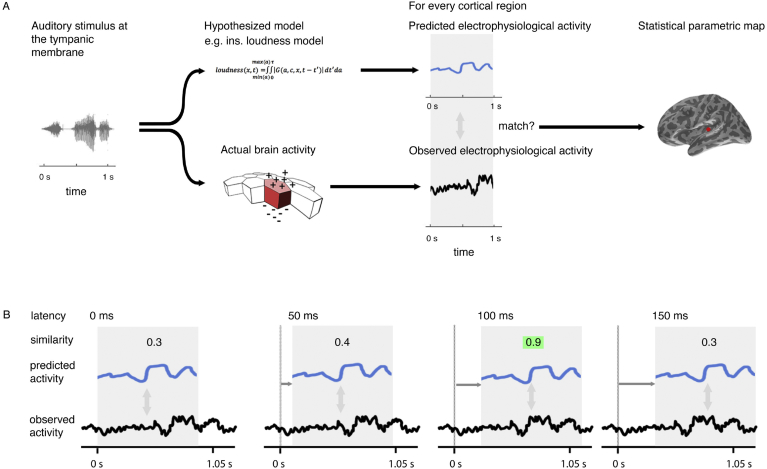
Technique overview. First (**A**), the electrophysiological activity of the brain in response to a given stimulus (measured using EMEG) is matched to the pattern of neural activity predicted by the model being evaluated. Predicted and observed activity are tested for similarity and the resulting statistical parametric map displays the regions (sources) where the match is statistically significant. Second (**B**), this procedure is repeated at different lags (illustrated here from 0 to 150 ms) between the onset of the observed neural activity and the onset of the predicted output. The similarity is highest at a specific lag (highlighted). This produces a statistical parametric map that changes over time.

The reconstructed distributed source current of the cortex is specified as the current of 10,242 cortical regions (sources), spaced uniformly over the cortex. The testing procedure involves examining each of these sources, looking for evidence that the current predicted by a model is similar to the current observed (Fig. 2A). This procedure is repeated at 5-ms intervals (Fig. 2B) across a range of time lags (−200 < *l* < 800 ms), covering the range of plausible latencies (0–800 ms) and a short, pre-stimulation range (−200 to 0 ms) during which we would expect to see no significant match (The 0–800 ms range was chosen because the study of Thwaites et al. (2015) showed little significant expression for instantaneous loudness after a latency of 500 ms; the 5-ms interval step was chosen because it is the smallest value that can be used given current computing constraints). This produces a statistical parametric map that changes over time as the lag is varied, revealing the evolution of similarity for a given model's predicted behavior with observed behavior over cortical location and time. Evidence of a model's similarity between its predicted behavior and cortical activity is expressed as a p-value, which is generated through the match-mismatch technique described in Thwaites et al. (2015), where evidence for similarity is described as significant if the p-value is less than a pre-defined value, α*. We refer to the observation of significant matches at a specific lag as ‘model expression’.

Setting α* so that it accurately reflects what is known about the data being tested can be difficult. In the current study, some of the measurements used in the tests are dependent on other measurements (because of spatial and temporal similarities between neighboring sources and lags). However, it is very difficult, if not impossible, to get accurate estimations of, for instance, the spatial dependencies between sources. In the present study, rather than accept assumptions about the dependencies that are hard to justify, we assumed that the data at each source and lag were independent (a ‘worst case’ scenario). As a result, the type II error rate is likely to be high, making the reported results ‘conservative’.

We used an exact formula for the familywise false-alarm rate to generate a ‘corrected’ α, α* of approximately 3 × 10^−13^ (see Thwaites et al., 2015, for the full reasoning); p-values greater than this are deemed to be not significant.

The results are presented as *expression plots*, which show the latency at which each of the 10,242 sources for each hemisphere best matched the output of the tested model (marked as a ‘stem’). The y-axis shows the evidence supporting the match at this latency: if any of the sources have evidence, at their best latency, indicated by a p-value lower than α*, they are deemed ‘significant matches’ and the stems are colored red, pink or blue, depending on the model.

**Fig. 3:**
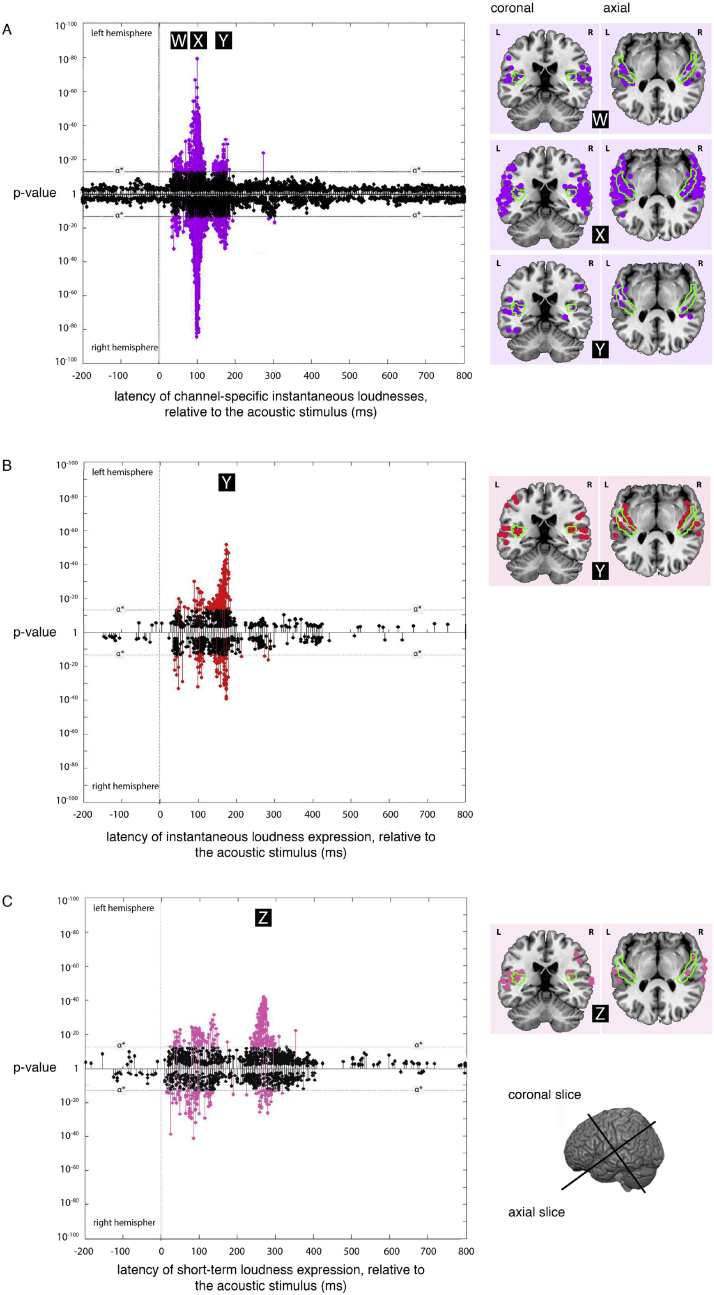
Expression plots for the *channel-specific instantaneous loudness, instantaneous loudness* and *short-term loudness* models. **(A)** Plot overlaying the expression of the nine *channel-specific instantaneous loudness* models across processing lags from −200 to +800 ms, relative to the acoustic signal. Results for the left and right hemispheres are plotted separately, with the right hemisphere plots inverted to allow comparison between hemispheres. The minimum p-values at a given source, over all latencies, are marked as ‘stems’. Stems at or above the stipulated value (p = 3 × 10^−13^) indicate significant expression of the *channel-specific instantaneous loudness* models that are not explained better by the other models tested. The cortical locations of significant sources at latencies W (45 ms), X (100 ms) and Y (165 ms) (labeled in black boxes) are indicated on the coronal and axial slices to the right of the plot. **(B)**. Plot of the expression for the overall *instantaneous loudness* model across processing lags from −200 to +800 ms, relative to the acoustic signal. Again, the cortical locations of significant sources at latency Y (165 ms) (labeled in black box) are indicated on the coronal and axial slices to the right of the plot. (**C)**. Plot of the expression for the *short-term loudness* model across processing lags from −200 to +800 ms, relative to the acoustic signal. The cortical locations of significant sources at latency Z (275 ms) (labeled in a black box) are indicated on the coronal and axial slices to the right of the plot. All three expression plots (with Fig. 6) implement models selected so that each source appears only once in the five plots. The boundary of the probabilistic anatomical parcellation of HG is shown in green (Rademacher et al., 1992; Fischl et al., 2004; Morosan et al., 2001). (For interpretation of the references to colour in this figure legend, the reader is referred to the web version of this article.)

The expression plots also allow us to chart which models are most likely at a particular source through a model selection procedure, using p-values as a proxy for model likelihood. For each model's expression plot we retain only those sources where the p-value is lower (has higher likelihood) than for the other models tested. Accordingly, each plot has fewer than 10,242 sources per hemisphere, but the five plots (Fig. 3, Fig. 6) taken together make up the full complement of 10,242 sources per hemisphere. It is important to note that this model selection procedure does not indicate that any one model is *significantly* better than another for some source. It indicates only that one model is better than another by some amount, even if the evidence may not differ strongly between models. We take this approach as we are only interested in the trend of which models explain the activity best in each source, and our aim is to distinguish between models that may be correlated over time.

In what follows, the input signal for all transformations is the estimated waveform at the tympanic membrane. This waveform was estimated by passing the digital representation of the signal waveform through a digital filter representing the effective frequency response of the earpieces used. This frequency response (called the transfer function) was measured using KEMAR KB0060 and KB0061 artificial ears, mounted in a KEMAR Type 45DA Head Assembly (G.R.A.S. Sound & Vibration, Holte, Denmark). This frequency response, measured as gain relative to 1000 Hz, was estimated to be ([*frequency*:*left gain*:*right gain*]): [125 Hz:−1.5:−0.8], [250 Hz:1.5:1.3], [500 Hz:1.5:2.6], [1000 Hz:0:0], [2000 Hz: 1.6: 0.5], [3000 Hz: −2.0:-0.5] [4000 Hz:−5.6:−3.7], [5000 Hz:−6.4:−5.1], [6000 Hz:−12.3:−13.3].

## 4 Methods and materials

The methods and materials are the same as those used in Thwaites et al. (2016). The reader is referred to that paper for full details.

### 4.1 Participants and stimuli

*Participants:* there were 15 right-handed participants (7 men, mean age = 24 years, range = 18–30). All gave informed consent and were paid for their participation. The study was approved by the Peterborough and Fenland Ethical Committee (UK). For reasons unconnected with the present study, all participants were native speakers of Russian.

*Stimuli*: A single 6 min 40 s acoustic stimulus (a Russian-language BBC radio interview about Colombian coffee) was used. This was later split in the analysis procedure into 400 segments of length 1000 ms. The stimulus was presented at a sampling rate of 44.1 kHz with 16-bit resolution. A visual black fixation cross (itself placed over a video of slowly fluctuating colors) was presented during the audio signal to stop the participant's gaze from wandering.

### 4.2 Procedure

Each participant received one practice stimulus lasting 20 s. Subsequent to this, the continuous 6 min 40 s stimulus was presented four times, with instructions to fixate on the cross in the middle of the screen while listening. After each presentation, the participant was asked two simple questions about the content of the stimulus, which they could answer using the button box. Having made a reply, they could rest, playing the next presentation when ready, again using the button box. Presentation of stimuli was controlled with Matlab, using the Psychophysics Toolbox extensions (Brainard, 1997, Pelli, 1997, Kleiner et al., 2007). The stimuli were binaurally presented at approximately 65 dB SPL via Etymotic Research (Elk Grove Village, Illinois) ER3 earpieces with 2.5 m tubes.

### 4.3 EMEG recording

Continuous MEG data were recorded using a 306 channel VectorView system (Elekta-Neuromag, Helsinki, Finland) containing 102 identical sensor triplets (two orthogonal planar gradiometers and one magnetometer) in a hemispherical array situated in a light magnetically shielded room. The position of the head relative to the sensor array was monitored continuously by four Head-Position Indicator (HPI) coils attached to the scalp. Simultaneous EEG was recorded from 70 Ag-AgCl electrodes placed in an elastic cap (EASYCAP GmbH, Herrsching-Breitbrunn, Germany) according to the 10/20 system, using a nose electrode as reference. Vertical and horizontal EOG were also recorded. All data were sampled at 1 kHz and were band-pass filtered between 0.03 and 330 Hz. A 3-D digitizer (Fastrak Polhemus Inc, Colchester, VA) recorded the locations of the EEG electrodes, the HPI coils and approximately 50–100 ‘headpoints’ along the scalp, relative to three anatomical fiducials (the nasion and left and right pre-auricular points).

### 4.4 Data pre-processing

Static MEG bad channels were detected and excluded from subsequent analyses (MaxFilter version 2, Elektra-Neuromag, Stockholm, Sweden). Compensation for head movements (measured by HPI coils every 200 ms) and a temporal extension of the signal–space separation technique (Taulu et al., 2005) were applied to the MEG data. Static EEG bad channels were visually detected and removed from the analysis (MNE version 2.7, Martinos Center for Biomedical Imaging, Boston, Massachusetts). The EEG data were re-referenced to the average over all channels. The continuous data were low-pass filtered at 100 Hz (zero-phase shift, overlap-add, FIR filtering). The recording was split into 400 epochs of 1000 ms duration. Each epoch included the 200 ms from before the epoch onset and 800 ms after the epoch finished (taken from the previous and subsequent epochs) to allow for the testing of different latencies. Epochs in which the EEG or EOG exceeded 200 ìV, or in which the value on any gradiometer channel exceeded 2000 fT/m, were rejected from both EEG and MEG datasets. Epochs for each participant were averaged over all four stimulus repetitions.

### 4.5 Source reconstruction

The locations of the cortical current sources were estimated using minimum-norm estimation [MNE] (Hämäläinen and Ilmoniemi, 1994, Gramfort et al., 2013, Gramfort et al., 2014), neuro-anatomically constrained by MRI images obtained using a GRAPPA 3D MPRAGE sequence (TR = 2250 ms; TE = 2.99 ms; flip-angle = 9°; acceleration factor = 2) on a 3T Tim Trio (Siemens, Erlangen, Germany) with 1-mm isotropic voxels. For each participant a representation of their cerebral cortex was constructed using FreeSurfer (Freesurfer 5.3, Martinos Center for Biomedical Imaging, Boston, Massachusetts). The forward model was calculated with a three-layer Boundary Element Model using the outer surface of the scalp and the outer and inner surfaces of the skull identified in the structural MRI. Anatomically-constrained source activation reconstructions at the cortical surface were created by combining MRI, MEG, and EEG data. The MNE representations were downsampled to 10,242 sources per hemisphere, roughly 3 mm apart, to improve computational efficiency. Representations of individual participants were aligned using a spherical morphing technique (Fischl et al., 1999). Source activations for each trial were averaged over participants. We employed a loose-orientation constraint (0.2) to improve the spatial accuracy of localization. Sensitivity to neural sources was improved by calculating a noise covariance matrix based on a 1-s pre-stimulus period. Reflecting the reduced sensitivity of MEG sensors for deeper cortical activity (Hauk et al., 2011), sources located on the cortical medial wall and in subcortical regions were not included in the analyses reported here.

### 4.6 Visualization

The cortical slices in Fig. 3 use the visualization software MRIcron (Georgia State Center for Advanced Brain Imaging, Atlanta, Georgia) with results mapped to the high-resolution colin27 brain (Schmahmann et al., 2000). The HG label in Fig. 5 is taken from the on *Desikan-Kilkenny-Tourville-40* cortical labelling atlas (Klein and Tourville, 2012), and the probabilistic anatomical parcellation of HG in Fig. 3 is based on the probabilistic atlases of Rademacher et al. (1992), Fischl et al. (2004) and Morosan et al. (2001).

## 5 Results

### 5.1 Channel-specific instantaneous loudness models

The regions where expression for one of the nine channel-specific instantaneous loudness models was the most significant of all the models tested, and below the α* threshold, were located mainly bilaterally at latencies of 45 ms, 100 ms and 165 ms (Fig. 3A).

**Fig. 4:**
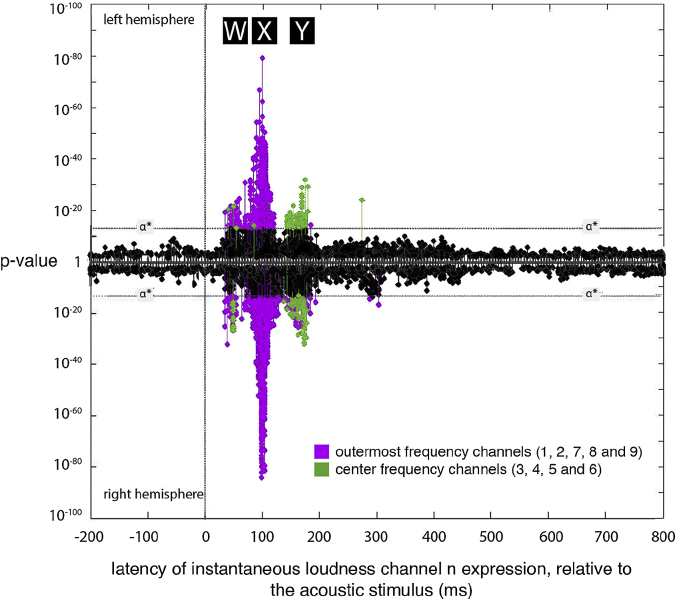
Expression plot illustrating the differences between the ‘center’ and ‘outermost’ instantaneous loudness channels. The figure reproduces data from Fig. 3A, but with expressions for the ‘center’ and ‘outermost’ instantaneous loudness channels (channels 3, 4, 5, 6 and 1, 2, 7, 8, 9, respectively) colored purple and green, respectively. The plot shows that expression at ‘W’ and ‘X’ (labeled in black boxes) is strongest for the outermost channels, while expression at ‘Y’ is strongest for the channels in the center. (For interpretation of the references to colour in this figure legend, the reader is referred to the web version of this article.)

This picture is complicated somewhat by the fact that significant expression was not found for all channels at all three latencies. Broadly speaking, the ‘center’ channels (that is, channels 3, 4, 5 and 6) were expressed at 165 ms, while the ‘outermost’ channels (1, 2, 7, 8 and 9) were expressed at 45 and 100 ms (Fig. 4).

**Fig. 5:**
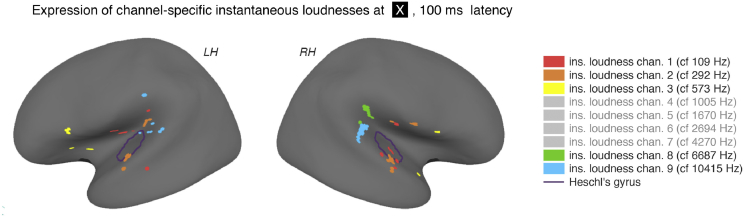
Positions of the expression for the channel-specific instantaneous loudnesses at 100 ms. The figure shows an inflated brain with the significant regions of entrainment to each of the nine tested instantaneous loudness channels, at 100 ms. Channels with no significant expression at this latency are ghosted in the key. For display purposes, only the top seven most significant vertices for each channels are shown. The boundary of HG, taken from the *Desikan-Kilkenny-Tourville-40* cortical labeling atlas (Klein and Tourville, 2012), is also shown.

The reported positions of the expression of these channels are uncertain as a consequence of the error introduced by the point-spread function inherent in EMEG source localization (see discussion). The expression of channels 1, 2, 3 and 5 at 45 ms appears to be bilateral, and dispersed loosely around the lateral sulcus, while the locations of the expression at 100 ms are found more clearly in or around HG (Fig. 5), although these are again somewhat dispersed.

The locations of the expression at 165 ms were bilateral, like the expression of the channels found at 45 ms, and dispersed loosely around the lateral sulcus (see accompanying cortical slices to the right of Fig. 3A). An interactive representation of these nine channels (and all models tested in this paper) can be viewed using The Kymata Atlas (2016).

### 5.2 Instantaneous loudness model

The regions where expression for the instantaneous loudness model was the most significant of the models tested, and below the α* threshold, were located mainly bilaterally with a latency of 165 ms (Fig. 3B). This expression was centered on the DLS.

### 5.3 Short-term loudness model

The regions where expression for the short-term loudness model was the most significant of the models tested, and below the α* threshold, were located mainly bilaterally with a latency of 275 ms (Fig. 3C). This expression was centered on the DLS and the superior temporal sulcus.

### 5.4 Hilbert envelope model

**Fig. 6:**
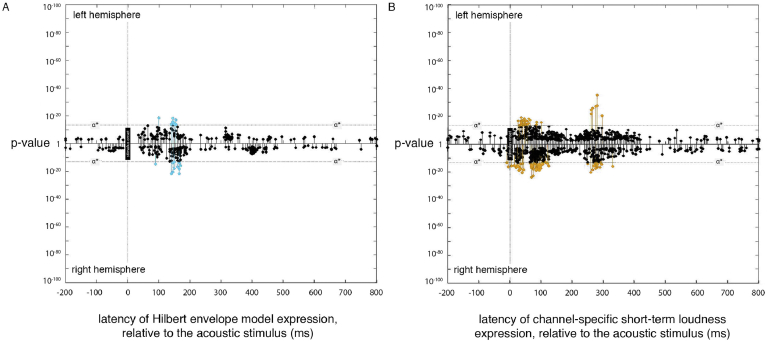
Expression plot for the *Hilbert envelope* and *channel-specific short-term loudness* model. (A) Plot of expression of the *Hilbert envelope* model (across processing lags from −200 to +800 ms, relative to the acoustic signal), showing the latencies of significant expression with the Hilbert model. (B) Plot of expression of the nine *channel-specific short-term loudness* models. All nine expression plots for each channel are overlaid into a single plot.

Small but significant expression was found for the Hilbert envelope model at 155 ms (Fig. 6A). The locations of this expression were similar to the locations of the sources entrained to instantaneous loudness. While significant, the evidence (in terms of p-values) for this expression was several orders of magnitude below those for the most significant instantaneous or short-term loudness expressions. The Hilbert envelope model is unrealistic, as it takes no account of the physical transformations occurring between the sound source and the cochlea or of the physiological transformations occurring in the cochlea. The entrainment found to the Hilbert envelope probably reflects its high correlation with the instantaneous loudness, and the noisiness of the data resulting from source estimation error.

### 5.5 Channel-specific short-term loudness models

Small but significant expression was found for the nine channel-specific short-term loudness models between 0 and 130 ms and around 300 ms (Fig. 6B). The locations of this expression were similar to the locations of the sources entrained to channel-specific instantaneous loudnesses. While significant, the evidence (in terms of p-values) for this expression was several orders of magnitude below those for the most significant instantaneous or short-term loudness expressions.

## 6 Discussion

The study of Thwaites et al. (2016) assessed entrainment to only three transforms: instantaneous loudness, short-term loudness and Hilbert envelope. As a result, it ascribed entrainment in HG and the DLS at 45, 100 and 165 ms latency to instantaneous loudness, and entrainment in the DLS and superior temporal sulcus at 275 ms latency as entrainment to short-term loudness, with little evidence of entrainment to the Hilbert envelope. The current study, which assessed entrainment to these three transforms plus eighteen new transforms, re-ascribes this early entrainment at 45 ms and 100 ms latency to the nine channel-specific instantaneous loudnesses. This supports the view that auditory information is split into frequency channels in the cochlea, this information is transmitted, in parallel, along the tonotopically organized afferent fibers making up the auditory nerve (Helmholtz, 1863, Fuchs, 2010, Meyer and Moser, 2010, von Bekesy, 1949), and that the information encoded in these channels reaches the cortex in separate cortical locations (Saenz and Langers, 2014).

The integration of these channels into a single ‘combined’ instantaneous loudness value appears to occur between 100 ms and 165 ms: expression for the channel-specific instantaneous loudnesses was predominantly found at 45 ms and 100 ms latency, and expression for overall instantaneous loudness was predominately found at 165 ms. This interpretation is, at first glance, seemingly contradicted by additional expression of channel-specific instantaneous loudness at 165 ms: if both channel-specific instantaneous loudness and instantaneous loudness are expressed at 165 ms, then there is no time for the conversion from the former to the latter. However, Fig. 4 shows that this 165 ms expression for channel-specific instantaneous loudness is likely a false positive, as it is mostly the central four channels (channels 3–6) that are associated with expression at this latency. The outputs of these four channels are, in this dataset, highly correlated with the overall instantaneous loudness (*ρ* = 0.9, 0.9, 0.8, and 0.6 for channels 3, 4, 5, and 6, respectively). As a result, it is difficult to assess whether this expression is primarily related to the four channel-specific instantaneous loudnesses or to the overall instantaneous loudness. The fact that the entrainment at 45 and 100 ms was largely to the outermost bands (which are mostly less correlated with overall instantaneous loudness (*ρ* = 0.5, 0.8, 0.4, −0.1, and −0.1 for channels 1, 2, 7, 8 and 9 respectively)) lends credence to the fact that the middle bands were also entrained at this time, and we assume this to be the case for the rest of this discussion. In future studies, we aim to reduce correlations between the outputs of these transforms by including naturalistic non-speech sounds (e.g. musical sounds, rain, running water etc.) in the stimulus.

The inherent insolvability of the inverse problem during EMEG source reconstruction (Grave de Peralta-Menendez et al., 1996, Grave de Peralta-Menendez and Gonzalez-Andino, 1998) means that significant ‘point spread’ of localization data was present, and the localization of entrainment (and the tonotopic map of the channel-specific instantaneous loudnesses in Fig. 6 in particular) should thus be treated with caution. However, evidence from other studies suggests that tonotopic mapping of frequency channels occurs in HG (see Saenz and Langers, 2014, for review), and the results found in this study are consistent with this. All expression at 100 ms latency (and, to a lesser extent, 45 ms) was found in or around HG, and the channels that showed entrainment in regions closer to HG (e.g. channels 1 and 2), had low (i.e. more significant) p-values. This is to be expected: where the localization is more accurate, the signal-to-noise ratio is presumably higher, resulting in lower p-values. Nevertheless, it is difficult to infer a tonotopic layout of CFs from the current data, beyond a possible low-frequency/anterior, high-frequency/posterior orientation (Fig. 5 *cf.* Baumann et al., 2013, Moerel et al., 2014, Saenz and Langers, 2014, Su et al., 2014). Improvements in source reconstruction (through the gathering of more data or improved inverse techniques) may reveal this layout more accurately.

**Fig. 7:**
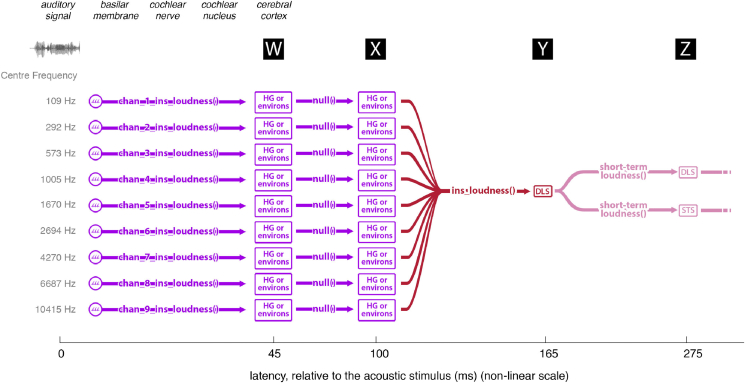
Implied pathway of loudness information. The figure illustrates one interpretation of the pathway suggested by the findings of this study, with latencies W, X, Y and Z (labeled in black boxes), corresponding to those same labels in Fig. 3A, B and C. First, the channel-specific instantaneous loudness transforms are applied, with the result of these transformations being entrained in or around HG at 45 ms (locations are approximate due to the source localization error). This information is then moved or copied (seemingly untransformed) to other areas located in HG or its environs (at 100-ms latency). The information in each of these channels is then combined to form instantaneous loudness, expressed in the DLS at 165 ms. From here, the instantaneous loudness is transformed to the short-term loudness, to be entrained in both the DLS and superior temporal sulcus (at 275 ms latency).

It cannot be determined from these results why the channel-specific instantaneous loudness information is ‘moved’ or ‘copied’ (i.e. with no intervening transform, signaled in Fig. 7 by a null() function) from 45 ms to 100 ms to a different region of the brain. Storage, or preparation for integration with other information, are both possibilities. Alternatively, one of these periods of entrainment may reflect entrainment to the output of a correlated, but untested, transform, perhaps one carried out as part of the construction of channel-specific instantaneous loudness.

Some evidence was found of entrainment to the channel-specific short-term loudnesses, but this evidence was several orders of magnitude below those for the most significant instantaneous or short-term loudness expressions. The weak significant entrainment to channel-specific short-term loudness may reflect a genuine effect or it may reflect false positives, since the channel-specific short-term loudness is correlated with the channel-specific instantaneous loudness for the same channel (*ρ* = 0.9, 0.8, 0.8, 0.8, 0.8, 0.8, 0.7, 0.8 and 0.7 for channels 1–9 respectively), and, for the middle channels, the channel-specific short-term loudness is correlated with the overall short-term loudness (*ρ* = 0.9, 0.8 and 0.8 for channels 3–5 respectively). Hence the data do not allow a clear conclusion about whether channel-specific instantaneous loudness is summed across channels to give the overall instantaneous loudness and the overall short-term loudness is determined from the overall instantaneous loudness, as assumed in the loudness model of Glasberg and Moore (2002), or whether short-term loudness is determined separately for each channel from the instantaneous loudness for that channel, and then short-term loudness values are summed across channels to give the overall short-term loudness, as assumed in the models of Chalupper and Fastl (2002) and Moore et al. (2016). The evidence from this study appears to favour the former.

Overall, these findings suggest a set of pathways whereby auditory magnitude information is moved through regions of the cortex (Fig. 7). Under this interpretation, the instantaneous loudness transform is determined in a number of frequency bands, each of which represents the information from a restricted region of the basilar membrane, with the output of this transformation being entrained in or around HG at 45 ms. This information is then moved or copied (seemingly untransformed) to other areas located in HG or its environs at a latency of 100 ms. From there, the information in each of these channels is combined, with the output of this transformation entrained in DLS at 165 ms. This instantaneous loudness is transformed to short-term loudness, to be entrained in both the DLS and superior temporal sulcus at 275 ms latency.

## 7 Conclusions

The results of this study suggest that the channel-specific instantaneous loudness transforms of incoming sound take place before 45 ms latency, and entrainment to the output of these transforms occurs in different regions of the cortex at latencies of 45 and 100 ms (bilaterally, in and around HG). Between 100 ms and 165 ms, these channel signals are combined, forming instantaneous loudness, entrained at 165 ms in DLS. The short-term loudness transform is then applied, with entrainment primarily at 275 ms, bilaterally in DLS and superior temporal sulcus. The locations of this entrainment are approximate due to the inherent error in source estimation of EMEG data. More work is needed to improve the accuracy of these reconstructions in order to improve the certainty of these locations.

## Figures and Tables

**Table 1:** The center frequencies of the nine channels in Hz and in Cams, and their respective KIDs for channel-specific instantaneous loudness.

Model name	Center frequency (Hz)	Center frequency (Cam)	Kymata ID
Instantaneous loudness channel 1	109	3.625	EUXJZ
Instantaneous loudness channel 2	292	7.625	PWMD2
Instantaneous loudness channel 3	573	11.625	5LHYD
Instantaneous loudness channel 4	1005	15.625	KAEKQ
Instantaneous loudness channel 5	1670	19.625	YYB73
Instantaneous loudness channel 6	2694	23.625	EN7SE
Instantaneous loudness channel 7	4270	27.625	UC4DR
Instantaneous loudness channel 8	6687	31.625	A8QRP
Instantaneous loudness channel 9	10,415	35.625	UJU6C
